# A rare case of lipoid pneumonia attributed to amiodarone

**DOI:** 10.1186/s41479-018-0056-3

**Published:** 2018-12-05

**Authors:** Ilektra Voulgareli, Alexandra Chronaiou, Dionisios Tsoukalas, George Tsoukalas

**Affiliations:** Department of Pneumonology, Sotiria Hospital, Athens, Greece

**Keywords:** Endogenous lipoid pneumonia, Amiodarone, Bronchoalveolar lavage, Prednisolone treatment

## Abstract

We report a case of endogenous lipoid pneumonia secondary to long-term use of amiodarone (> 30 years) for atrial fibrillation in a 76-year-old Caucasian woman, presenting with cough and dyspnea. Endogenous Lipoid pneumonia is a rare underdiagnosed condition more prevalent in adults. It is usually asymptomatic and a diagnosis is generally made in patients who have become clinically unstable or when an abnormal lung shadow is found on a chest X-ray. In the case here described it was diagnosed by fiberoptic bronchoscopy with bronchoalveolar lavage (BALF) where fat-laden macrophages (oil red O stain) were identified. Since a history of use of oil-based products had been ruled out, amiodarone was deemed to be the most likely cause of lipoid pneumonia. The patient was managed with the replacement of amiodarone with digoxin and treated with oral prednisolone. The patient has remained clinically stable with radiological improvement during a follow-up of two years.

## Introduction

Endogenous lipoid pneumonia is an uncommon clinical condition, more prevalent in adult age, but generally underdiagnosed. It can be associated with bronchial obstruction [1], systemic diseases such as rheumatoid arthritis, Hodgkin’s lymphoma, and Wegener’s granulomatosis or it can be idiopathic [2]. Here we report the case of a 76-year-old woman who was diagnosed with endogenous lipoid pneumonia attributed to chronic use of amiodarone (30 years) and successfully treated with discontinuation of amiodarone and prescription of prednisolone. Amiodarone was replaced with digoxine . Most reported cases of lipoid pneumonia diagnosed as exogenous lipoid pneumonia are due to either aspiration or inhalation of mineral or vege oils [3, 11]. Lipoid pneumonia occurs mostly in the elderly and in children with developmental disabilities, presumably because these groups are more prone to aspiration [15].

## Case report

A 76-year-old Caucasian female attended our pulmonology outpatient clinic with a four-week history of cough with mucous-purulent sputum and dyspnea. The patient was a nonsmoker and had worked for a few years in a cotton factory. Her past medical history was negative for any contact with substances known to be associated with lipoid pneumonia. She had not travelled recently and had no pets. She had been treated for atrial fibrillation with amiodarone (200 mg OD) for 30 years, systemic hypertension, diabetes mellitus type II and hypothyroidism. Physical examination revealed a well built and nourished patient with pulse rate 69 per minute and blood pressure 110/70 mmHg. Vital signs were within normal range with SpO2 of 95% in room air. Her physical examination revealed bibasal lung crackles, but no evidence of pallor, icterus, cyanosis, clubbing or lymphadenopathy. Her blood work-up was within normal range. Pulmonary function testing demonstrated moderate restrictive lung disease and a decreased diffusion capacity. (see Table 1).

**Table 1:** Selected pulmonary function test results

	Observed	Predicted	Predicted (%)
Forced vital capacity (FVC)	2.32 L	2.59 L	90
Forced expiratory volume in 1 s (FEV1)	1.27 L	1.97 L	65
FEV1/FVC (%)	73	76	96
Inspiratory capacity	1.49 L	2.04 L	73
Total lung capacity	4.22 L	4.75 L	89
Residual volume	1.97 L	2.13 L	92
DLCO corrected for hemoglobin	10.71	19.48	55

Tests of pulmonary function indicating moderate restrictive pulmonary disease and a marked defect diffusing capacity. There was no improvement with inhaled bronchodilators (data not shown)

*DLCO* Diffusing capacity of the lung for carbon monoxide

**Fig. 1:**
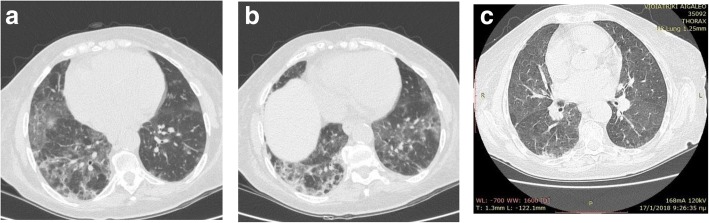
Computed tomography images of a 76-year-old female with lipoid pneumonia revealed ground-glass opacities and bronchiectasis in both lungs (**a**), (**b**). The shadows improved 2 years later (**c**)

Since chest X-ray showed bilateral infiltrates, a high resolution computed tomography (HRCT) was conducted. The latter revealed areas of ground glass appearance significantly in the lower lobes of both lungs and airspace consolidations were seen as well (Fig.1, [a], [b]). The shadows improved 2 years later (Fig. 1, [c]).

Radiologically, differential diagnosis included atypical pneumonia, interstitial lung disease and tuberculosis was ruled out. The patient underwent fiberoptic bronchoscopy for bronchial wash and bronchoalveolar lavage (BALF) was collected for immunological studies as well. The bronchial washings were sent for smear for acid fast bacilli and cytological examination. No microorganisms were isolated by 48bacteriological examination and no malignant cells were found.

**Fig. 2:**
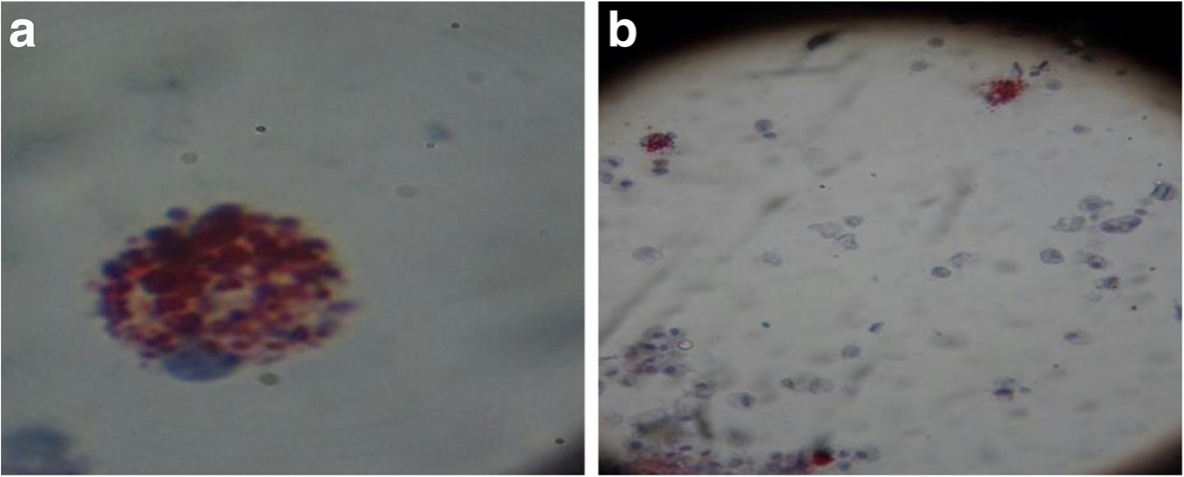
**a**, **b** The bronchoalveolar fluid examination in a 76-year-old female with lipoid pneumonia. Oil phagocytosis by alveolar macrophages was observed under a microscope with Oil Red staining

The total cell count of the BALF was 287,500/ml. The cells consisted of macrophages (64%), lymphocytes (31%), neutrophils (3%), and eosinophils (2%). The evaluation of BALF with specific staining and coloration showed the presence of fat-laden macrophages (oil red O stain). Figure 2 [a], [b] Extracellular oily droplets were found on a sputum cytology examination. These findings were suggestive of lipoid pneumonia.

The patient had never aspirated or inhaled mineral or vegetable oils and she had never used petroleum jelly intranasally or extra-nasally as a decongestant. However, it is described in the literature that chronic use of amiodarone can cause endogenous lipoid pneumonia (phospholipidosis) [17]. Amiodarone, as an amphiphilic cationic compound, interferes with the movement of phospholipids across intracellular membranes and inhibits phospholipid catabolism through its potent inhibitory effect on lysosomal phospholipase 2. Drug-induced phospholipidosis assumes the form of a ‘foamy cell ‘response.

In our case, the patient had been taking amiodarone (200 mg OD) for 30 years which was considered the most likely cause. It was therefore discontinued and replaced with digoxin (0.25 mg OD) for the treatment of atrial fibrillation. She was also started on oral prednisolone (20 mg OD) which was gradually tapered over a period of six months.

## Discussion

Lipoid pneumonia can be classified into endogenous lipoid and exogenous lipoid pneumonia [2, 5, 6]. Compared to exogenous lipoid pneumonia, endogenous lipoid pneumonia is rarer and its etiology, which is not clear, may be associated with metabolic or secretory abnormalities of cholesterol in the alveolar epithelial tissue and excessive release of lipids uptaken by histiocytes [2]. It is considered a chronic foreign body reaction to fat, characterized by lipid-laden macrophages [5] and is usually associated with bronchial obstruction [1]. Additionally, endogenous lipoid pneumonia can be associated with systemic diseases such as rheumatoid arthritis, Hodgkin’s lymphoma, and Wegener’s granulomatosis. It can also be idiopathic and the pathogenes is unknown [2]. In the present case report there was no history of use of oil-based products so a diagnosis of endogenous lipoid pneumonia was more likely in the absence of systemic diseases or obstructive changes on the imaging of the lungs. Since it is described in the literature that chronic use of amiodarone can cause endogenous lipoid pneumonia it was deemed that this could be the agent responsible for the patient’s condition [15].

Amiodarone and its metabolites can produce lung damage directly through a cytotoxic effect and indirectly via an immunological reaction. Amiodarone may induce the production of toxic oxygen radicals, which can directly damage cells. In addition, amiodarone appears to promote the accumulation of phospholipids in tissues. There is hyperplasia of type II pneumocytes and a widening of alveolar septae with a cellular inflammatory infiltrate and varying degrees of interstitial fibrosis [18]. On light microscopy, vacuolization of the cytoplasm was seen in alveolar pneumocytes, bronchial epithelial cells and endothelial cells [16]. The typical features and diagnosis of the lipoid pneumonia depend on the presence of lipid-laden macrophages (the so-called ‘foamy cells’) in respiratory samples such as sputum, bronchoalveolar lavage fluid (BALF) or fine-needle aspiration (FNA) cytology/biopsy from lung lesions and of course on a high index of suspicion [2, 4, 7–9].

The usual course includes an insidious onset and non-specific respiratory symptoms such as dyspnea and/or cough. Rarely, it may present itself as an acute respiratory illness [6, 7, 9, 10] even with hemoptysis [13].

Radiological findings are diverse and may mimic carcinoma, acute or chronic pneumonia, ARDS, or a localized granuloma. High-resolution computed tomography (HRCT) is the best imaging modality for the diagnosis of lipoid pneumonia. However, the radiological manifestations of the disease can be indistinguishable from pneumonia, lung cancer or/ and interstitial lung diseases [12]. In the literature it has also been reported that CT scan and magnetic resonance imaging can detect fat within pulmonary tissue. The most commonly described feature is alveolar consolidations of low attenuation values, ground glass opacities with a thickening of intralobular septa (‘crazy-paving’ pattern), or alveolar nodules [2, 6–8, 11].

Treatment of lipoid pneumonia is not well studied and experiences are only limited to case reports. Some authors have suggested the use of systemic corticosteroids in order to slow the inflammatory response.

Nevertheless, corticosteroids may not be used routinely and may only be used if the lung injury is severe and ongoing. Some authors have described resection of the most involved lung segments while others the use of immunoglobins or whole lung-lavages [2, 4, 7–9]. Our patient responded well to oral corticosteroids and her clinical conditions improved during a follow-up of two years.

## Conclusion

In conclusion, endogenous lipoid pneumonia is very uncommon and in this case the diagnosis was attributed to the long-term use of amiodarone.

Lipoid pneumonia was confirmed by BALF (bronchoalveolar lavage) where foamy macrophages positive in Oil-red-O staining were found [14]. Withdrawal of the suspected agent and a trial of oral corticosteroids were the mainstay of our treatment. If undetected, lipoid pneumonia can insidiously or symptomatically lead to pulmonary fibrosis and end-stage lung disease.

## Abbreviations

ARDS: Acute Respiratory Distress Syndrome
BALF: bronchoalveolar lavage
DLCO: Diffusing capacity of the lung for carbon monoxide
FNA: Fine Needle Aspiration
HRCT: High Resolution Computed Tomography
